# Serum Heme Oxygenase-1 and BMP-7 Are Potential Biomarkers for Bone Metabolism in Patients with Rheumatoid Arthritis and Ankylosing Spondylitis

**DOI:** 10.1155/2016/7870925

**Published:** 2016-05-26

**Authors:** Tong-ling Yuan, Jin Chen, Yan-li Tong, Yan Zhang, Yuan-yuan Liu, James Cheng-Chung Wei, Yi Liu, Yi Zhao, Martin Herrmann

**Affiliations:** ^1^Department of Rheumatology and Immunology, West China Hospital, Sichuan University, Chengdu 610041, China; ^2^Department of Health Statistics, West China School of Public Health, Sichuan University, Chengdu 610041, China; ^3^Division of Allergy, Immunology and Rheumatology, Chung Shan Medical University Hospital, Taichung 40201, Taiwan; ^4^Institute of Medicine, Chung Shan Medical University, Taichung 40201, Taiwan; ^5^Institute of Integrative Medicine, China Medical University, Taichung 40201, Taiwan; ^6^Department of Internal Medicine 3, University of Erlangen-Nuremberg, 91054 Erlangen, Germany

## Abstract

*Backgrounds*. Heme oxygenase-1 (HO-1) has been reported to play a regulatory role in osteoclastogenesis. Bone morphogenetic protein (BMP) pathways induce osteoblastic differentiation and bone remodeling.* Aims*. To identify serum levels of HO-1, BMP-7, and Runt related-transcription factor 2 (Runx2) in patients with rheumatoid arthritis (RA) and ankylosing spondylitis (AS) and to investigate the relationships between HO-1, BMP-7, Runx2, and other common biomarkers for bone metabolism.* Results*. Serum levels of HO-1 and BMP-7 were revealed to be significantly higher in patients with RA or AS than in healthy controls (*p* < 0.01). In RA group, HO-1 was positively correlated with BMP-7, Runx2, and tartrate-resistant acid phosphatase-5b (TRAP-5b) (*p* < 0.05, resp.), BMP-7 was positively correlated with Runx2 and TRAP-5b (*p* < 0.05, resp.), and Runx2 was negatively correlated with N-terminal midfragment of osteocalcin (NMID) (*p* < 0.05). In AS group, we observed identical correlation between HO-1 and BMP-7, but opposite correlations between BMP-7 and TRAP-5b and between Runx2 and NMID, when comparing with the RA cohort.* Conclusion*. Our findings suggest that HO-1 and BMP-7 are potential biomarkers for bone metabolism in patients with RA and AS. The different correlations between the bone markers point to distinct differences in bone remodeling pathways in the two types of arthritis.

## 1. Introduction

Chronic inflammatory arthritis involves both inflammation and, consequently, disruption to bone architecture, but the predominant locations and patterns of structural change differ between different types of rheumatic disease [1, 2]. Rheumatoid arthritis (RA) is characterized by synovitis, interaction of lymphocytes, macrophages, and synovial cells, the production of matrix metalloproteinases and cytokines such as tumor necrosis factor (TNF), interleukin-1 (IL-1), and interleukin-6 (IL-6), leading to excessive erosion of articular cartilage and marginal bone, and defective bone repair [2–6]. While ankylosing spondylitis (AS), the prototype of spondyloarthritis, is manifested by enthesitis, excessive and ectopic ossification leads to syndesmophytes, along with bone loss [1–3]. To date, some proinflammatory cytokines such as TNF, IL-23, and IL-17 and factors including transforming growth factor (TGF*β*) and bone morphogenetic proteins (BMPs) could promote such pathologic process in AS. To maintain structural integrity of bone, osteoclasts resorb bone and osteoblasts produce osteoid which is subsequently mineralized to new bone [2, 3, 7]. Unbalanced activity between osteoclasts and osteoblasts resulting in dysregulated bone remodeling occurs both in RA and AS [3]. Therapies that reduce inflammation suppress the bone resorption but insufficiently repair the erosions in RA or impede the ongoing ectopic bone formation in AS [8]. The mechanisms of regulation of bone metabolism in arthritis are still insufficiently understood.

Heme oxygenase-1 (HO-1), the rate-limiting enzyme in heme catabolism, catalyses the degradation of heme into free iron (Fe^2+^), biliverdin, and carbon monoxide (CO) [9, 10]. It has been well documented that the cytoprotective roles of HO-1 include anti-inflammatory, antioxidant, and antiapoptotic functions. There is emerging evidence that HO-1 is involved in bone metabolism. HO-1 has the ability to downregulate inflammation in RA and osteoarthritis (OA), regulating osteoclastogenesis and bone resorption in RA, limiting cartilage degradation, and enhancing repair in OA [10–12]. It also downregulates the senescence responses in OA articular tissues [9, 13]. Research on serum HO-1 in RA and AS is scarce.

BMPs are members of the TGF*β* superfamily. The BMP pathways play a major part in early osteoblast differentiation and the formation of tendon and ligament. BMPs lead to bone and joint remodeling and maintenance of bone mass in the mature skeleton. BMPs induce marrow stromal cells to differentiate toward osteoblasts and chondrocytes, by enhancing the expression of osteogenesis-driving transcription factors such as Runt related-transcription factor 2 (Runx2) and osterix and that of bone matrix components (alkaline phosphatase, osteocalcin, and type I collagen). BMP-7 has been demonstrated to strongly induce osteogenesis of arthritis in many studies [14–16]. Runx2, the downstream factor of several BMPs, is assumed to be a key regulator of osteoblast differentiation. Runx2 promotes the expression of bone matrix protein especially during the early stage of osteoblasts, but inhibits the maturation of osteoblasts [17]. However, some studies demonstrated the opposite function of both BMPs and Runx2 that regulate osteoclasts.

In the current study, we intended to assess serum levels of HO-1, BMP-7, Runx2 in RA and AS and the relationships between these and further common markers of bone metabolism in RA and AS. Our data suggest that HO-1 and BMP-7, of which the serum levels were significantly elevated, are candidate biomarkers for bone metabolism in patients with RA and AS. The correlative relationships between the bone markers identify distinct differences in the pathways for bone remodeling in patients with AS and RA.

## 2. Materials and Methods

### 2.1. Patients

All patients who were evaluated in the Department of Rheumatology and Immunology, West China Hospital, Sichuan University (April 2014–July 2015), were consecutively invited to participate in this study. They are all Chinese. All RA patients met the 2009 ACR/EULAR criteria, and all AS patients met the 1984 modified New York criteria. Exclusion criteria were osteomalacia, psoriasis, inflammatory bowel disease, recent infarction, currently serious infection, other concomitant rheumatic diseases, other serious diseases, and maternity. Healthy controls were recruited volunteers, who had no known diseases, in West China Hospital of Sichuan University. The Ethics Committee of Sichuan University approved the study and informed consent was obtained from each participant. Medical records were subsequently reviewed to obtain information on demographic characteristic, smoking history, disease duration, clinical manifestation, current or previous treatment, and laboratory index which included NMID, BALP, CTX, TRAP-5b, and so forth.

### 2.2. Clinical Assessments

To assess disease activity and severity, Disease Activity Score (DAS) 28 was evaluated for RA patients, and the Bath Ankylosing Spondylitis Disease Activity Index (BASDAI), Functional Index (BASFI), and Metrology Index (BASMI) were used for AS patients. The score of DAS28 above 3.2 in RA or that of BASDAI above 4 in AS was considered as high or moderate disease activity. All clinical assessments were evaluated by well-trained nurses and rheumatologists.

### 2.3. Measurements of HO-1, BMP-7, and Runx2

Serum samples were collected from all subjects at the same time with the measurements of clinical and laboratory parameters and were stored at −80°C until assayed. Enzyme-linked immunosorbent assays (ELISAs) for human HO-1, BMP-7, and Runx2 were performed according to the protocol of manufacturer (Cloud-Clone Corp., USA). The minimum detectable dose was typically less than 0.124 ng/mL for HO-1, <0.093 ng/mL for BMP-7, and <0.054 ng/mL for Runx2, respectively. Absorbance was read at 450 nm in BioTek spectrophotometer.

### 2.4. Statistical Analysis

Statistical analyses were performed by SPSS 19.0 for Windows. Since most continuous variables in the study were skewed in distribution, descriptive statistics were presented as median and interquartile range (IQR); in addition, the comparisons of demographic and biochemical data between groups were mostly performed using nonparametric statistics (Kruskal-Wallis *H* test for 3 independent group comparisons, followed by Dunn's multiple comparisons test). The comparison of categorical variables was performed by chi-square test. Correlations between two variables were calculated by Spearman's correlation. *p* value less than 0.05 was considered statistically significant, if not addressed, while the type I error in post hoc comparisons was corrected by the Bonferroni rule.

## 3. Results

### 3.1. Characteristics of the Patients

In total, 40 RA patients, 35 AS patients, and 20 healthy controls were enrolled in this study. The demographic and clinical characteristics of RA and AS patients were described in Table 1, and the demographic characteristics of healthy controls were described in Table S1 in Supplementary Material available online at http://dx.doi.org/10.1155/2016/7870925. A significant female and old age predominance was noted in the RA group, as expected, compared with AS group or the controls. The sexes and median ages in the AS group were similar to the healthy control group, while more females and older ages were found in the RA group (Table S1). The median DAS28-ESR score of RA patients was 4.3 and the median BASDAI score of AS patients was 3.0, indicating that most of them were at a moderate disease activity.

### 3.2. Levels of Biomarkers among the Study Groups

**Figure 1:**
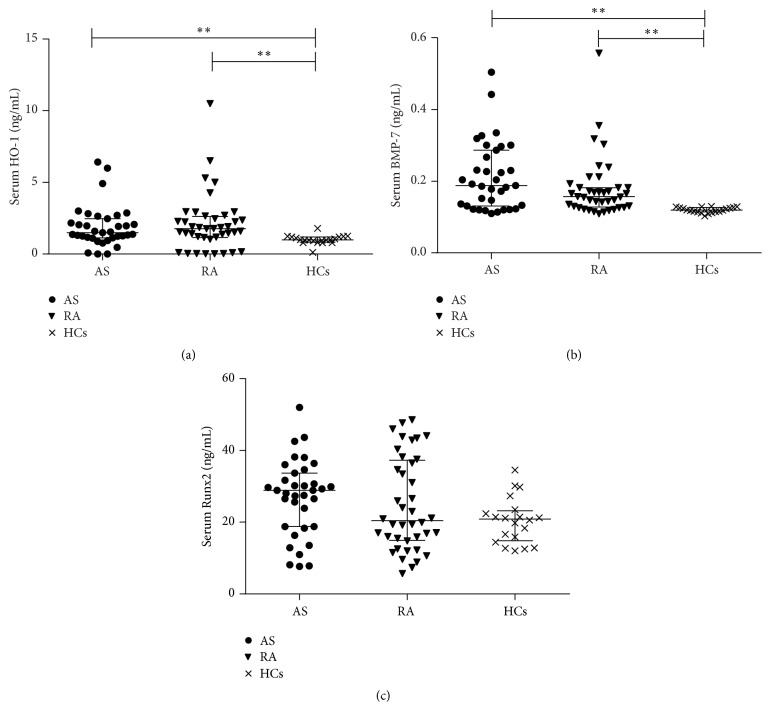
(a) Serum HO-1 levels were, respectively, higher in RA patients and AS patients than those in HCs. (b) Serum BMP-7 levels were, respectively, higher in RA patients and AS patients than those in HCs. (c) Serum Runx2 levels were not significantly higher either in RA patients or in AS patients than those in HCs. ^*∗∗*^ *p* < 0.01.

We observed significantly higher median levels of HO-1 in serum of RA patients (1.77 ng/mL) and AS patients (1.49 ng/mL) in comparison to controls (0.99 ng/mL, *p* < 0.01, resp., Figure 1(a)). There were higher serum levels of HO-1 in RA than AS, but the difference was not statistically significant (*p* = 1.00). Increased levels of BMP-7 were detected in patients with RA (0.16 ng/mL) and AS (0.19 ng/mL) when compared with the healthy controls (0.12 ng/mL, *p* < 0.001, resp., Figure 1(b)). BMP-7 did not differ significantly between the patients with RA and AS (*p* = 0.586). The median levels of Runx2 in the RA group (20.42 ng/mL) were lower than those of the healthy controls (20.87 ng/mL) and the patients with AS (28.90 ng/mL), but all the differences did not reach statistical significance (*p* > 0.05, Figure 1(c)). The median levels of NMID in RA patients were significantly lower than that in AS patients (*p* = 0.001, Table S1). For the serum levels of other bone metabolism biomarkers (BALP, CTX, and TRAP-5b), no statistical significance has been found among the three cohorts (*p* > 0.05, Table S1).

### 3.3. Correlation between HO-1, BMP-7, Runx2, and Other Biomarkers in Patients with RA and AS

In the RA group, HO-1 was positively correlated with BMP-7 (*p* = 0.012), Runx2 (*p* = 0.010), and TRAP-5b (*p* = 0.034), respectively, but not with the other bone metabolism biomarkers. BMP-7 was positively correlated with Runx2 (*p* = 0.026) and TRAP-5b (*p* < 0.001), and Runx2 was negatively correlated with NMID (*p* < 0.05) (Table 2). Similarly, HO-1 was positively correlated with BMP-7 (*p* = 0.002) in the AS group, but not with Runx2, NMID, BALP, CTX, or TRAP-5b. In contrast to the RA group, a negative correlation between BMP-7 and TRAP-5b (*p* = 0.023) and a positive correlation between Runx2 and NMID (*p* = 0.007) were to be seen in the AS group (Table 2).

### 3.4. Correlation of HO-1, BMP-7, and Runx2 with Clinical Parameters in Patients with RA and AS

In the RA group, serum levels of HO-1, BMP-7, and Runx2 were all not significantly correlated with ESR, CRP, and DAS28-ESR. In the AS group, serum HO-1 and BMP-7 levels showed no significant correlation with ESR, CRP, BASDAI, BASFI, or BASMI, and only serum Runx2 levels were significantly associated with BASMI scores (*p* = 0.003) (Table 3).

## 4. Discussion

As we know, bone erosion with inadequate bone formation is a central feature of RA; conversely, new bone formation and modest erosive osteopenia are a major feature of AS [18, 19]. However, the cellular and molecular mechanisms of bone remodeling are elusive. This fact strongly limits the improvement of prognosis for patients with RA and AS.

In this study, we observed significantly elevated serum levels of HO-1 and BMP-7 in patients with RA or AS compared to healthy controls. Moreover, HO-1 was positively correlated with BMP-7, Runx2, and TRAP-5b, BMP-7 was positively correlated with Runx2 and TRAP-5b, and Runx2 was negatively correlated with NMID in patients with RA. Nevertheless, HO-1 was merely positively correlated with BMP-7 in patients with AS. In contrast to patients with RA, patients with AS showed negative and positive correlations between BMP-7 and Runx2 and between Runx2 and NMID, respectively.

HO-1 plays a crucial role in cytoprotection. Some studies described increased levels of HO-1 in the synovial tissue, synovial fluid, and synovial cells of patients with RA compared to those in controls [4, 20, 21]. Data on serum levels of HO-1 in patients with RA or AS are scarce. The upregulation of HO-1 has been shown to inhibit local osteoclastogenesis in TNF transgenic mice with arthritis [10]. Guillén et al. revealed that HO-1 induction not only inhibited cartilage degradation but also enhanced proteoglycan and collagen II synthesis of OA chondrocytes [12]. According to our results, increased levels of HO-1 support the prior findings that HO-1 possibly prevented the osteoclastogenesis in both RA and AS.

As we know, BMPs are well established as key regulators of osteoblasts biology. The activity of BMP pathways not only induces mesenchymal progenitor cell differentiation into osteoblasts by enhancing osteogenesis-driving transcription factors such as Runx2 and Osterix but also regulates mineral deposition by enhancing the expression of alkaline phosphatase (ALP), type I collagen, and noncollagen proteins such as osteocalcin and bone sialoprotein in mature osteoblasts [2, 22]. Expressions of further BMP family members (BMP-2, BMP-4, and BMP-7) were reportedly increased at sites of enthesitis in a mouse model of SpA and at the Achilles tendon in biopsies of early-stage humans enthesopathy [23]. In accordance with these histological specimens, as well as previous studies on serum levels of BMP-7 in RA and AS, our findings demonstrated the high systemic expression of BMP-7 in patients with RA and AS [14, 15]. Though research on serum levels of BMP-7 in patients with RA was rare, these data support the view that BMP-7 might participate in the pathogenesis of RA, and serum BMP-7 might also predict osteoproliferation in RA. Besides, BMPs can promote osteoclasts differentiation as well as activity via the RANKL/OPG pathway. Previous studies have revealed that osteoclasts expressed BMP and were directly influenced by BMP [16, 24]. Moreover, Maurer et al. observed that BMP-7 inhibited the differentiation of human CD14+ monocytes to osteoclasts [25].

Runx2 is one of the target genes of BMPs. Most previous studies on cell and molecular levels of Runx2 or BMP/Runx2 pathway have demonstrated its regulatory function of bone development and extracellular matrix. Runx2 mainly induces the synthesis of major bone matrix during early-stage osteoblast differentiation. The function of Runx2 can be enhanced and inhibited by several diverse molecules [26]. Runx2 is regulated by fibroblast growth factors (FGFs), retinoic acid, 1*α*,25(OH)_2_D_3_, and TNF-*α*, besides BMPs [26]. Earlier genetic study performed by Grcevic et al. reported that the gene level of Runx2 was significantly decreased and increased versus healthy controls in patients with RA and AS, respectively [27]. There was no significant difference of serum levels of Runx2 among the three cohorts in our study. The inconsistent results from our finding and others may be due to the differences in experimental approaches, the selection bias, the therapeutic interferences, or the Runx2 being regulated by some factors. In addition, Runx2 induces the differentiation of monocytes to osteoclasts [17]. Much of those theories came from cell cultures or animal models of arthritis.

Bone consolidation correlates well with serum levels of biochemical bone markers like BALP, type I collagen, and osteocalcin [28]. NMID, the serum N-terminal midfragment of osteocalcin, which is released from bone matrix, appears to be more reliable than osteocalcin to evaluate osteoblast function and can induce the differentiation and activity of osteoclasts [29, 30]. Osteocalcin is expressed in mature osteoblasts [17] and can also be upregulated by BMP-2/Smad/Runx2 pathway [31]. Some isoforms of Runx2, such as Runx2wt and Runx2Δ7, might upregulate the expression of osteocalcin in osteoblasts [32]. Tartrate-resistant acid phosphatase (TRAP) of osteoclasts was found to be a crucial marker of bone resorption [3, 33]. TRAP-5b, an isoform of TRAP, is more specific and sensitive as a bone resorption marker.

HO-1 was positively correlated with BMP-7, Runx2, and TRAP-5b, respectively; and BMP-7 was positively correlated with Runx2 in the RA cohort. These results revealed that HO-1 might participate in the BMP-7/Runx2 pathway to promote osteoproliferation, meanwhile involve in compensatory prevention of osteoclastogenesis in RA. HO-1 was merely positively correlated with BMP-7, and BMP-7 was not correlated with Runx2 in AS. A possible explanation may be that HO-1 participated in other BMP-7 pathways to promote osteoproliferation. Further studies on HO-1 or BMP pathways directly regulating osteoblast function and mineralization in patients with RA and AS are warranted. On the other hand, BMP-7 was positively correlated with TRAP-5b in the RA group, supporting the hypothesis that BMP-7 induces osteoclasts in RA. On the contrary, negative correlation between BMP-7 and TRAP-5b in patients with AS supports another hypothesis that BMP-7 inhibits osteoclasts in AS. The opposite effect conforms to the pathological features that RA manifests excessive osteoclastogenesis; conversely, AS manifests obvious osteoblastogenesis [5]. Furthermore, there are strong interferences of age, sex, smoking, disease duration, and medications. Interestingly, we found that Runx2 was negatively correlated with NMID in RA patients but positively correlated with NMID in AS patients. The possible discrepancy for the correlation between RA and AS could be explained by the complex connection between Runx2 and bone remodeling. The expression and activity of Runx2 were inhibited or enhanced by some factors, and the distinct isoforms of Runx2 determined the synthesis of NMID. Our observation in humans might confirm most previous concepts from cell cultures or animal models of arthritis that most bone markers take on double-edged effects, contributing to bone formation or bone resorption. These results indicated the distinct molecular mechanisms of bone remodeling in patients with RA and AS. Even more important, the same bone marker might exert paradoxical effects on the distinct prototype arthritis. To the best of our knowledge, this is the first investigation of these pairs of correlations between bone markers in RA and AS.

None of serum levels of HO-1, BMP-7, and Runx2 was associated with inflammatory markers or disease activity in any of the patient groups under investigation. In contrast to our data, Kitamura et al. found that HO-1 levels in RA synovial fluids correlated with serum levels of CRP [4]. A previous study on either RA or OA revealed that HO-1 significantly reduced the production of proinflammatory cytokines [4, 9, 13]. In line with our results, Korkosz et al. found no correlation between the serum BMP-7 concentrations and either ESR or CRP in patients with AS [34]. The discrepancies between our and others' results on HO-1 might be due to the effects of the disease spectra and states as well as the complex interactions of various cytokines and biomarkers. In this study, HO-1, BMP-7, and Runx2 might show an independent correlation to bone remodeling. And Runx2 merely positively associated with BASMI in the AS group, indicating that Runx2 might reflect the spinal mobility.

Although very interesting, our study had some limitations. For instance, this study was a cross section design with relatively small sample size, and the biomarkers examined were probably affected by the medications and the severe disease states. Some critical values thus might result from the small sample size. Furthermore, the female predominance of RA patients might contribute to a potential bias when comparing the biomarkers with patients suffering from AS and healthy controls. A further longitudinal and large sample study employing additional approaches is required to validate the findings of this preliminary study.

## 5. Conclusions

In summary, we detected increased serum levels of HO-1 and BMP-7 in patients with RA or AS. Moreover, serum levels of HO-1, BMP-7, and Runx2 showed significant correlations with other common bone markers, but no correlation with Disease Activity Scores in RA and AS. Our observation, taken together with the other studies discussed above, indicates that HO-1 and BMP-7 have high potential to contribute to the basic pathological processes involved in the pathogenic bone metabolism in RA and AS. Furthermore, the same bone marker might exert paradoxical effects on the distinct prototype arthritis; the different correlations between the bone markers point to distinct differences in the pathways for bone remodeling in RA and AS.

## Supplementary Material

Serum levels of biomarkers in patients with rheumatoid arthritis (RA), ankylosing spondylitis (AS) and in the controls. Data were given as median (IQR). Data compared by Kruskal-Wallis H test, followed by Dunn's multiple comparisons test, except sex by chi-square test. A p value < 0.0167 is considered significant after Bonferroni correction for multiple testing. p values were comparisons among RA, AS and Control samples. ^&^Significant difference compared between RA and AS (p < 0.05, data not shown). n.s.: the variables were included in analysis, but not significantly different between the three groups by Kruskal-Wallis H test. IQR: interquartile range; NMID: N-terminal mid-fragment of osteocalcin; BALP: bone alkaline phosphatase; CTX: C-terminal telopeptide of type I collagen; TRAP-5b: Tartrate-resistant acid phosphatase-5b.

## Figures and Tables

**Table 1:** Characteristics of patients with rheumatoid arthritis (RA) and ankylosing spondylitis (AS) in southwest China. Data were given as median (IQR).

Characteristics	Number of Patients (%) or median (IQR)	
	RA, *n* = 40	AS, *n* = 35
Female/male	32/8	8/27
Age, yrs	42 (37, 49)	37 (29, 44)
Present or past smokers (%)	6 (15.0)	24 (68.6)
Symptom duration, yrs	1 (1, 3)	5 (2, 10)
CRP, mg/L	6.9 (2.7, 12.8)	13.1 (6.8, 22.4)
ESR, mm/h	32.0 (15.0, 67.0)	46.5 (28.0, 72.8)
DAS28-ESR score	4.3 (3.4, 5.2)	NA
BASDAI score	NA	3.0 (2.4, 4.3)
BASFI score	NA	1.7 (0.8, 3.2)
BASMI score	NA	1.0 (0, 3.0)

IQR: interquartile range; NA: not applicable; CRP: C-reactive protein; ESR: erythrocyte sedimentation rate; DAS28-ESR: Disease Activity Scores 28 using ESR; BASDAI: Bath Ankylosing Spondylitis (BAS) Disease Activity Index; BASFI: BAS Functional Index; BASMI: BAS Metrology Index.

**Table 2:** Correlation between HO-1, BMP-7, Runx2, and other biomarkers in patients with RA and AS. Correlations were performed using Spearman's correlation coefficient (*r*_*s*_).

	RA (*r*_*s*_)			AS (*r*_*s*_)		
	HO-1	BMP-7	Runx2	HO-1	BMP-7	Runx2
HO-1		0.392^*∗*^	0.403^*∗∗*^		0.499^*∗∗*^	−0.029
BMP-7	0.392^*∗*^		0.352^*∗*^	0.499^*∗∗*^		−0.131
Runx2	0.403^*∗∗*^	0.352^*∗*^		−0.029	−0.131	
NMID	0.007	0.067	−0.328^*∗*^	−0.023	−0.272	0.447^*∗∗*^
BALP	0.201	0.116	−0.115	−0.249	−0.319	0.097
CTX	0.247	0.209	−0.069	0.106	−0.255	0.328
TRAP-5b	0.337^*∗*^	0.562^*∗∗*^	0.134	−0.127	−0.384^*∗*^	0.301

^*∗*^ *p* < 0.05; ^*∗∗*^ *p* < 0.01. RA: rheumatoid arthritis; AS: ankylosing spondylitis; HO-1: heme oxygenase-1; BMP-7: bone morphogenetic protein-7; Runx2: Runt related-transcription factor 2; NMID: N-terminal midfragment of osteocalcin; BALP: bone alkaline phosphatase; CTX: C-terminal telopeptide of type I collagen; TRAP-5b: tartrate-resistant acid phosphatase-5b.

**Table 3:** Correlation between serum levels of HO-1, BMP-7, and Runx2 and measurements of disease activity and severity in patients with RA and AS. Correlations were performed using Spearman's correlation coefficient (*r*_*s*_).

	RA (*r*_*s*_)			AS (*r*_*s*_)				
	ESR, mm/h	CRP, mg/L	DAS28-ESR score	ESR, mm/h	CRP, mg/L	BASDAI score	BASFI score	BASMI score
ESR, mm/h		0.607^*∗∗*^	0.833^*∗∗*^		0.480^*∗∗*^	0.361^*∗*^	0.580^*∗∗*^	0.284
CRP, mg/L	0.607^*∗∗*^		0.552^*∗∗*^	0.480^*∗∗*^		0.437^*∗∗*^	0.579^*∗∗*^	0.398^*∗*^
HO-1, ng/mL	−0.068	−0.049	−0.118	−0.205	−0.068	−0.163	−0.197	−0.156
BMP-7, ng/mL	0.030	−0.096	0.001	−0.026	−0.203	0.023	−0.059	−0.119
Runx2, ng/mL	0.010	0.186	−0.048	0.051	0.209	0.103	0.286	0.483^*∗∗*^

^*∗*^ *p* < 0.05; ^*∗∗*^ *p* < 0.01. RA: rheumatoid arthritis; AS: ankylosing spondylitis; ESR: erythrocyte sedimentation rate; CRP: C-reactive protein; HO-1: heme oxygenase-1; BMP-7: bone morphogenetic protein-7; Runx2: Runt related-transcription factor 2; DAS28-ESR: Disease Activity Scores 28 using ESR; BASDAI: Bath Ankylosing Spondylitis (BAS) Disease Activity Index; BASFI: BAS Functional Index; BASMI: BAS Metrology Index.
