# Supplementary material for: Serum Heme Oxygenase-1 and BMP-7 Are Potential Biomarkers for Bone Metabolism in Patients with Rheumatoid Arthritis and Ankylosing Spondylitis
Source: Biomed Res Int. 2016 May 26;2016:7870925. doi: 10.1155/2016/7870925 (PMC4899581; doi:10.1155/2016/7870925)
Supplement: Supplementary file 1 — Serum levels of biomarkers in patients with rheumatoid arthritis (RA), ankylosing spondylitis (AS) and in the controls. Data were given as median (IQR). Data compared by Kruskal-Wallis H test, followed by Dunn's multiple comparisons test, except sex by chi-square test. A p value < 0.0167 is considered significant after Bonferroni correction for multiple testing. p values were comparisons among RA, AS and Control samples. &Significant difference compared between RA and AS (p < 0.05, data not shown). n.s.: the variables were included in analysis, but not significantly different between the three groups by Kruskal-Wallis H test. IQR: interquartile range; NMID: N-terminal mid-fragment of osteocalcin; BALP: bone alkaline phosphatase; CTX: C-terminal telopeptide of type I collagen; TRAP-5b: Tartrate-resistant acid phosphatase-5b. [file 7870925.f1.doc]

**TABLE S1:** Serum levels of biomarkers in patients with rheumatoid arthritis (RA), ankylosing spondylitis (AS) and in the controls. Data were given as median (IQR).

|  |  | Controls, n = 20 |  | RA, n = 40 | |  | AS, n = 35 | |
| --- | --- | --- | --- | --- | --- | --- | --- | --- |
|  |  | Median (IQR) |  | Median (IQR) | *p* |  | Median (IQR) | *p* |
| female/male |  | 9/11 |  | 32/8**^&^** | **0.006** |  | 8/27**^&^** | 0.087 |
| Age, yrs |  | 31, (26, 36) |  | 42 (37, 49)**^&^** | **< 0.001** |  | 37 (29, 44)**^&^** | 0.209 |
| NMID, ng/ml |  | 15.00 (13.4, 19.4) |  | 13.30 (9.7, 20.0)**^&^** | 0.810 |  | 18.60 (15.5, 27.4)**^&^** | 0.127 |
| BALP, ug/ml |  | 15.00 (13.2, 16.9) |  | 13.74 (11.5, 18.1) | n.s. |  | 17.90 (12.4, 20.5) | n.s. |
| CTX, ng/ml |  | 0.36 (0.3, 0.4) |  | 0.42 (0.3, 0.6) | n.s. |  | 0.48 (0.3, 0.7) | n.s. |
| TRAP-5b, U/L |  | 3.51 (2.8, 4.2) |  | 2.61 (1.8, 3.6) | 0.061 |  | 3.26 (2.5, 4.1) | 1.000 |

Data compared by Kruskal-Wallis H test, followed by Dunn’s multiple comparisons test, except sex by chi-square test. A p value < 0.0167 is considered significant after Bonferroni correction for multiple testing. *p* values were comparisons between RA/AS and Control samples. **^&^** Significant difference compared between RA and AS (*p* < 0.05, data not shown). n.s.: the variables were included in analysis, but not significantly different between the three groups by Kruskal-Wallis H test. IQR: interquartile range; NMID: N-terminal mid-fragment of osteocalcin; BALP: bone alkaline phosphatase; CTX: C-terminal telopeptide of type I collagen; TRAP-5b: Tartrate-resistant acid phosphatase-5b.
